# Associations of beta-catenin alterations and MSI screening status with expression of key cell cycle regulating proteins and survival from colorectal cancer

**DOI:** 10.1186/1746-1596-8-10

**Published:** 2013-01-21

**Authors:** Sakarias Wangefjord, Jenny Brändstedt, Kajsa Ericson Lindquist, Björn Nodin, Karin Jirström, Jakob Eberhard

**Affiliations:** 1 Department of Clinical Sciences, Division of Pathology, Lund University, Skåne University Hospital, Lund, 221 85, Sweden; 2 Department of Clinical Sciences, Division of Oncology, Lund University, Skåne University Hospital, Lund, 221 85, Sweden

## Abstract

**Background:**

Despite their pivotal roles in colorectal carcinogenesis, the interrelationship and prognostic significance of beta-catenin alterations and microsatellite instability (MSI) in colorectal cancer (CRC) needs to be further clarified. In this paper, we studied the associations between beta-catenin overexpression and MSI status with survival from CRC, and with expression of p21, p27, cyclin D1 and p53, in a large, prospective cohort study.

**Methods:**

Immunohistochemical MSI-screening status and expression of p21, p27 and p53 was assessed in tissue microarrays with tumours from 557 cases of incident CRC in the Malmö Diet and Cancer Study. Chi Square and Spearman’s correlation tests were used to explore the associations between beta-catenin expression, MSI status, clinicopathological characteristics and investigative parameters. Kaplan-Meier analysis and Cox proportional hazards modelling were used to assess the relationship between beta-catenin overexpression, MSI status and cancer specific survival (CSS).

**Results:**

Positive MSI screening status was significantly associated with older age, female sex, proximal tumour location, non-metastatic disease, and poor differentiation, and inversely associated with beta-catenin overexpression. Beta-catenin overexpression was significantly associated with distal tumour location, low T-stage and well-differentiated tumours. Patients with MSI tumours had a significantly prolonged CSS in the whole cohort, and in stage III-IV disease, also in multivariable analysis, but not in stage I-II disease. Beta-catenin overexpression was associated with a favourable prognosis in the full cohort and in patients with stage III-IV disease. Neither MSI nor beta-catenin status were predictive for response to adjuvant chemotherapy in curatively treated stage III patients. P53 and p27 expression was positively associated with beta-catenin overexpression and inversely associated with MSI. Cyclin D1 expression was positively associated with MSI and beta-catenin overexpression, and p21 expression was positively associated with MSI but not beta-catenin overexpression.

**Conclusions:**

Findings from this large, prospective cohort study demonstrate that MSI screening status in colorectal cancer is an independent prognostic factor, but not in localized disease, and does not predict response to adjuvant chemotherapy. Beta-catenin overexpression was also associated with favourable outcome but not a treatment predictive factor. Associations of MSI and beta-catenin alterations with other investigative and clinicopathological factors were in line with the expected.

**Virtual slides:**

The virtual slides for this article can be found here: http://www.diagnosticpathology.diagnomx.eu/vs/8778585058652609

## Background

Colorectal cancer (CRC) is the third most common cancer in men and the second most common cancer in women worldwide. More than 1,2 million new cases are diagnosed globally every year, with more than 600,000 related deaths in 2008 [1].

Colorectal carcinogenesis is a complex multistage process involving multiple genetic alterations and at least three distinct pathogenetic pathways have been characterized: chromosomal instability (CIN), microsatellite instability and CpG island methylator phenotype (CIMP) [2,3].

The chromosomal instability pathway (CIN) is characterized by karyotypic abnormalities, e.g. aneuploidy and loss of heterozygosity, and certain mutations including activation of K-ras and inactivation of APC, loss of p53, and loss of heterozygosity for the long arm of chromosome 18. This pathway accounts for 65-70% of sporadic CRC [4,5] and for cancers associated with familial adenomatous polyposis (FAP), constituting less than 1% of all CRC [6].

Beta-catenin is a membrane-associated protein with essential functions in the regulation of cellular adhesion and as the major mediator of the Wnt-signaling pathway. When Wnt-receptors are activated, kinases in the APC complex are inhibited, leading to accumulation of cytoplasmic beta-catenin and its translocation to the nucleus. This facilitates the transcription of various target genes, e.g. cyclin D1, hence contributing to tumour progression [7-11].

Despite its crucial role in colorectal carcinogenesis, previous studies on altered beta-catenin expression as a prognostic marker or predictor of chemotherapy response in CRC have been conflicting [12-15].

The second pathway is initiated by germline mutations in the mismatch repair (MMR) genes, e.g. *MLH1*, *MSH2*, *MSH6*, and *PMS2*, or somatic tumour MLH1 promoter methylation, leading to microsatellite instability (MSI). MSI is detected in 15-20% of all cases of CRC, predominantly tumours located in the proximal colon, and in almost all tumours from patients with hereditary nonpolyposis colon cancer (HNPCC), accounting for 3-5% of all CRC [16,17].

CRC patients with MSI tumours seem to have a significantly better prognosis compared to those with MSS in most studies, but might not benefit from adjuvant fluorouracil (FU) treatment [16-22]. On a molecular level, there is in vitro data supporting that an intact mismatch repair system is required to induce apoptosis of FU-modified DNA [23].

Finally, the CpG island methylator phenotype (CIMP) provides a third pathway to CRC through predisposition to epigenetic DNA hypermethylation, causing transcriptional silencing of tumour supressor genes [24,25].

The aim of the present study was to examine the prognostic and treatment predictive significance of beta-catenin overexpression and MSI screening status in tumours from a large prospective CRC cohort (n = 557). A secondary aim was to examine the associations of beta-catenin alterations and MSI status with key cell cycle regulators p53, p21, p27 and cyclin D1. In a previous study on tumours from this cohort, elevated cyclin D1 expression was found to associate with a prolonged survival, in particular in male patients [26].

## Methods

### Study group

The Malmö Diet and Cancer Study (MDCS) is a population-based prospective cohort study with the primary aim to examine whether a Western diet rich in fat and low in fruit and vegetables increases the risk of certain forms of cancer. Between 1991–1996, a total number of 30 446 individuals; 12 120 (39.8%) men and 18 326 (60.2%) women between 44–74 years where enrolled (from a background population of 74 138) [27]. Until 31 Dec 2008, 626 incident cases of CRC had been registered in the MDCS. Cases were identified from the Swedish Cancer Registry up until 31 Dec 2007, and from The Southern Swedish Regional Tumour Registry for the period of 1 Jan - 31 Dec 2008. All tumours with available slides or paraffin blocks were histopathologically re-evaluated on haematoxylin and eosin stained slides. Histopathological, clinical and treatment data were obtained from the clinical and/or pathology records. TNM staging was performed according to the American Joint Committee on Cancer (AJCC). Information on vital status and cause of death was obtained from the Swedish Cause of Death Registry up until 31 Dec 2009. Follow-up started at date of diagnosis and ended at death, emigration or 31 Dec 2009, whichever came first. None of the CRC cases registered until 31 Dec 2008 was lost due to emigration during follow-up. Median follow-up time was 3.35 years (range 0–17.69) for the full cohort (n = 626) and 6.05 years (range 1.03-17.69) for patients alive (n = 344). Patient and tumour characteristics of the cohort have been described in detail previously [26,28,29]. Ethical permission was obtained from the Ethics Committee at Lund University for the MDCS (Ref. 51/90), and the present study (Ref. 530/2008).

### Tissue microarray construction

Cases with an insufficient amount of tumour material were excluded, whereby a total number of 557 (89.0%) tumours were suitable for tissue microarray (TMA) construction. Areas representative of cancer were marked on haematoxylin & eosin stained slides and TMAs were constructed as previously described [26,28]. In brief, two 1.0 mm cores were taken from each tumour and mounted in a new recipient block using a semi-automated arraying device (TMArrayer, Pathology Devices, Westminster, MD, USA). As demonstrated previously, there was no selection bias regarding the distribution of clinicopathological characteristics between the TMA cohort and the full cohort [28].

### Immunohistochemistry and staining evaluation

For immunohistochemical analysis, 4 μm TMA-sections were transferred to glass slides (Menzel Super Frost Plus), dried at room temperature, and baked in a heated chamber for 2 hours at 60°C. Deparaffinization and antigen retrieval was performed using the PT Link system (DAKO, Glostrup, Denmark) in a high pH buffer. Staining was performed in an Autostainer Plus (DAKO) with monoclonal antibodies against p21 (clone SX118, DAKO) diluted 1:25, p27 (clone SX53G8, DAKO) diluted 1:100, and p53 (clone DO-7, DAKO) diluted 1:100, using a di-amino-benzidine (DAB) based visualization kit (K801021-2, Dako Denmark A/S). Counterstaining was performed using Mayer’s hematoxylin. Immunohistochemical staining and evaluation of cyclin D1 expression had been performed previously using a monoclonal anti-cyclin D1 antibody DSC-6 (DAKO) diluted 1:50 [26]. Nuclear expression of p21, p27 and cyclin D1 was evaluated in a similar fashion, taking both the fraction of positive cells and staining intensity into account. Specifically, the nuclear staining intensity was denoted as 0 (negative), 1 (weak), 2 (moderate) or 3 (strong), and the proportion of positive tumour nuclei as 0 (0 to 1%), 1 (2 to 25%), 2 (26 to 50%), 3 (51 to 75%) and 4 (> 75%). p53 positivity was defined as > = 50% tumour cells with strong nuclear staining intensity in acordance with previous studies [30].

For definition of MSI screening status, immunohistochemical staining for MLH1, PMS2, MSH2 and MSH6 was performed as previously described [29]. Immunohistochemical stainings were evaluated as negative when all tumour cells showed loss of nuclear staining. Surrounding stromal cells and tumour infiltrating lymphocytes served as internal controls for each biopsy core. A nuclear reaction of tumour cells was assessed as a positive staining. MSI screening status was evaluated using monoclonal antibodies against MLH1 (Clone ES05, DAKO, diluted 1 : 100), PMS2 (Clone A16–4, 556415, BD Pharmingen, San Diego, CA, USA, diluted 1 : 300), MSH2 (Clone FE11, NA27, Calbiochem, San Diego, CA, USA, diluted 1 : 100), and MSH6 (EPR3945, Epitomics, Burlingame, CA, USA, diluted 1 :100) as previously described [31]. Immunohistochemical stainings were evaluated as negative when all tumour cells showed loss of nuclear staining. Surrounding stromal cells and tumour infiltrating lymphocytes served as internal controls for each biopsy core. Any nuclear reaction of tumour cells was assessed as positive staining. Tumour samples lacking nuclear staining of MLH1, PMS2, MSH2 or MSH6 were considered to have a positive MSI screening status, and referred to as MSI, and tumours with negative MSI screening status are referred to as MSS.

Immunohistochemical staining of beta-catenin was performed with a monoclonal anti-beta-catenin antibody (# 610153 BD Pharmingen, San Diego, Ca, USA), diluted 1:5000. The staining was evaluated as previously described [32,33], whereby membranous staining was denoted as 0 (present) or 1 (absent), cytoplasmic staining intensity as 0–2 and nuclear staining intensity as 0–2. The total score ranging from 0 (corresponding to membrane staining only, as in normal colonic mucosa) to 5 (tumours with strong nuclear and cytoplasmic staining) was then divided into three categories; 1 = score 0–1, 2 = score 2–3 and 3 = score 4–5).

All immunohistochemical stainings were evaluated by two independent observers (SW and KJ), who were blinded to clinical and outcome data. Scoring differences were discussed in order to reach consensus.

### Statistical analysis

Chi Square and Spearman’s correlation (R) tests were used to explore the associations between clinicopathological and investigative parameters. Kaplan-Meier analysis and log rank test were used to illustrate differences in cancer specific survival (CSS) according to beta-catenin overexpression and MSI screening status. Cox regression proportional hazards models were used for estimation of hazard ratios (HRs) for death from CRC in both uni- and multivariable analysis, adjusted for age, gender, T-stage, N-stage, M-stage, differentiation grade and vascular invasion. A backward conditional selection method was used for variable selection by the model. All tests were two-sided. A p-value of 0.05 was considered significant. All statistical analyses were performed using IBM SPSS Statistics version 20.

## Results

### Associations of MSI screening status and beta-catenin overexpression with clinicopathological characteristics and investigative factors

**Figure 1:**
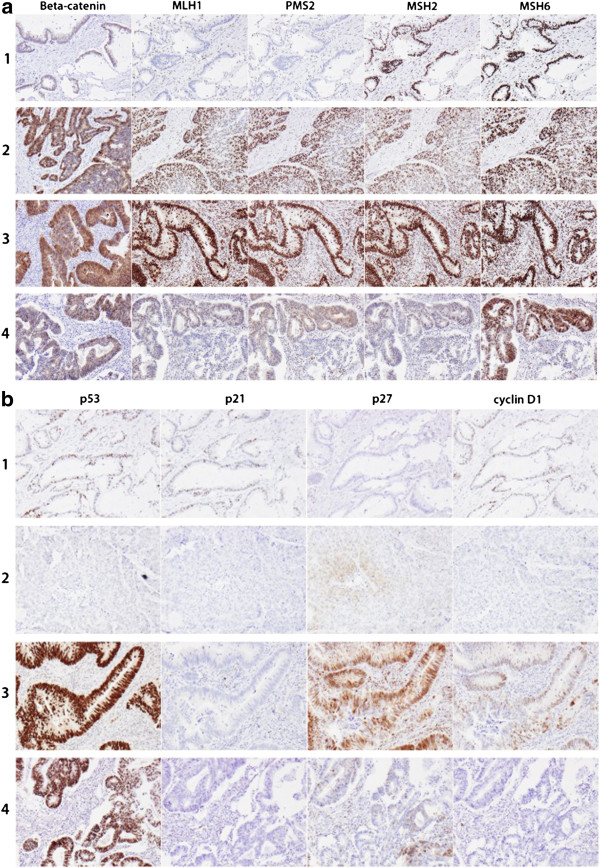
**Distribution of the investigative markers in four cases.** Immunohistochemical images from four different cases. 1. Female patient, age 70, with T3N0M0 tumour in transverse colon; beta-catenin grade 1 (0-1-0), MSI, p53 negative, p21 = 2/1, p27 negative, cyclin D1 = 2/2. 2: Female patient, age 77, with T4N1M1 tumour in descendent colon; beta-catenin grade 3 (0-1-2), MSS, p53 negative, p21 negative, p27 2/2, cyclin D1 1/1. 3. Male patient, age 75, with T4NXM tumour in proximal colon; beta-catenin grade 5 (1-2-2), MSS, p53 positive, p21 negative, p27 4/3, cyclin D1 3/2. 4. Male patient, age 68, with T2N2M0 rectal cancer; beta-catenin grade 3 (1-1-1), MSS; p53 positive, p21 negative, p27 3/3, cyclin D1 negative. **A**: staining of beta-catenin (membrane-cytoplasm-nucleus) and mismatch repair proteins MLH1, PMS2, MSH2 and MSH6. **B**: staining of p53, p21 (fraction/intensity), p27 (fraction/intensity) and cyclin D1 (fraction/intensity).

Representative immunohistochemical images of the investigative markers in tumours from four different cases are shown in Figure 1A & B.

As seen in Table 1 there were significant associations between MSI and older age, female sex, proximal tumour location, non metastatic disease (N0,M0) and low differentiation grade. Further, beta-catenin expression was associated with distal tumour location, less advanced T-stage and intermediate or high tumour differentiation grade.

**Table 1:** Associations of MSI screening status and beta-catenin expression with clinicopathological characteristics

	**MSI screening status**			**Beta-catenin score**			
**n (%)**	**MSS**	**MSI**	**P-value**	**0-1**	**2-3**	**4-5**	**P-value**
	**438(85.0)**	**77(15.0)**		**159(30.2)**	**170(32.3)**	**198(37.6)**	
**Age**							
<=75	308(70.3)	41(53.2)	0.003*	101(63.5)	123(72.4)	133(67.2)	0.561
>75	130(29.7)	36(46.8)		58(36.5)	47(27.6)	65(32.8)	
**Sex**							
Female	222(50.7)	50(64.9)	0.021*	87(54.7)	90(52.9)	100(50.5)	0.425
Male	216(49.3)	27(35.1)		72(45.3)	80(47.1)	98(49.5)	
**Tumour location**							
Proximal	107(24.5)	62(80.5)	<0.001**	84(53.2)	50(29.4)	37(18.8)	<0.001**
Transverse	12(2.8)	8(10.4)		11(7.0)	7(4.1)	5(2.5)	
Descending	26(6.0)	1(1.3)		6(3.8)	11(6.5)	9(4.6)	
Sigmoid	105(24.1)	3(3.9)		19(12.0)	31(18.2)	60(30.5)	
Rectum	186(42.7)	3(3.9)		38(24.1)	71(41.8)	86(43.7)	
Unknown	2	0		1	0	1	
**T stage**							
1	45(10.8)	3(3.9)	0.116	12(8.0)	11(6.9)	23(11.9)	0.039*
2	53(12.7)	7(9.2)		14(9.3)	21(13.1)	27(14.0)	
3	257(61.5)	54(71.1)		99(66.0)	100(62.5)	119(61.7)	
4	63(15.1)	12(15.8)		25(16.7)	28(17.5)	24(12.4)	
missing	20	1		9	10	5	
**N stage**							
0	222(56.5)	54(72.0)	0.017*	84(57.5)	86(55.8)	109(60.9)	0.249
1	101(25.7)	12(16.0)		28(19.2)	44(28.6)	44(24.6)	
2	70(17.8)	9(12.0)		34(23.3)	24(15.6)	26(14.5)	
missing	45	2		13	16	19	
**M stage**							
0	352(81.7)	72(93.5)	0.010*	123(78.8)	140(83.3)	166(85.1)	0.131
1	79(18.3)	5(6.5)		33(21.2)	28(16.7)	29(14.9)	
missing	7	0		3	2	3	
**Differentiation grade**							
High	28(6.5)	6(8.0)	0.003**	16(10.3)	8(4.8)	10(5.1)	0.016*
Intermediate	321(74.5)	40(53.3)		94(60.3)	115(69.3)	159(81.5)	
Low	82(19.0)	29(38.7)		46(29.5)	43(25.9)	26(13.3)	
missing	7	2		3	4	4	
**Vascular invasion**							
No	121(47.8)	26(57.8)	0.220	48(50.5)	33(36.3)	68(58.1)	0.177
Yes	132(52.2)	19(42.2)		47(49.5)	58(63.7)	49(41.9)	
missing	185	32		64	79	81	

Columnar percentage in parentheses. χ2-test of association was used for 2x2 tables and χ2-test for linear trend for tables with more than two rows and/or columns. The categories marked as missing were not included in the analysis. *Significant at the 0.05 level. **Significant at the 0.01 level. N1 = 1-3 positive nodes, N2= > 4 positive nodes. MSS = Microsatellite stable, MSI = Microsatellite unstable. Proximal colon = appendix, cecum, ascending colon, hepatic flexure; descending colon = splenic flexure and descending colon.

Associations between beta-catenin overexpression, MSI screening status and investigative factors are shown in Table 2. MSI screening status was inversely associated with beta-catenin overexpression. MSI correlated significantly to cyclin D1 expression (fraction and intensity), p21 expression (fraction and intensity), and inversely with p53 expression and p27 expression (fraction and intensity). Beta-catenin overexpression correlated significantly to p53 expression, cyclin D1 expression (fraction), and p27 expression (fraction and intensity).

**Table 2:** Associations of MSI screening status and beta-catenin expression with expression of p21, p27 and p53

	**MSI screening status**			**Beta-catenin score**			
**n (%)**	**MSS**	**MSI**	**P-value**	**0-1**	**2-3**	**4-5**	**P-value**
	**438(85.0)**	**77(15.0)**		**159(30.2)**	**170(32.3)**	**198(37.6)**	
**P53 status**							
Negative	196(40.0)	62(80.5)	<0.001**	101(65.2)	92(55.1)	76(38.6)	<0.001**
Positive	230(54.0)	15(19.5)		54(34.8)	75(44.9)	121(61.4)	
missing	12	0		4	3	1	
**Cyclin D1 fraction**							
0-1%	94(22.1)	2(2.6)	<0.001**	36(23.4)	36(21.7)	31(15.8)	0.001**
2-25%	159(37.4)	26(34.2)		63(40.9)	65(39.2)	63(32.1)	
26-50%	65(15.3)	16(21.1)		20(13.0)	29(17.5)	33(16.8)	
51-75%	87(20.5)	25(32.9)		27(17.5)	32(19.3)	53(27.0)	
>75%	20(4.7)	7(9.2)		8(5.2)	4(2.4)	16(8.2)	
missing	13	1		5	4	2	
**Cyclin D1 intensity**							
Negative	94(22.1)	2(2.6)	<0.001**	36(23.4)	36(21.7)	31(15.8)	0.085
Weak	154(36.2)	20(26.3)		51(33.1)	59(35.5)	68(34.7)	
Moderate	129(30.4)	41(53.9)		49(31.8)	55(33.1)	68(34.7)	
Strong	48(11.3)	13(17.1)		18(11.7)	16(9.6)	29(14.8)	
missing	13	1		5	4	2	
**P27 fraction**							
0-1%	54(12.7)	24(31.2)	0.003**	43(27.9)	33(19.8)	8(4.1)	<0.001**
2-25%	96(22.6)	14(18.2)		36(23.4)	43(25.7)	35(17.9)	
26-50%	90(21.2)	16(20.8)		32(20.8)	40(24.0)	36(18.4)	
51-75%	134(31.5)	15(19.5)		31(20.1)	36(21.6)	83(42.3)	
>75%	51(12.0)	8(10.4)		12(7.8)	15(9.0)	34(17.3)	
missing	13	1		5	3	2	
**P27 intensity**							
Negative	54(12.7)	24(31.2)	<0.001**	43/27.9)	33(19.8)	8(4.1)	<0.001**
Weak	96(22.6)	22(28.6)		46(29.9)	39(23.4)	37(18.9)	
Moderate	142(33.4)	18(23.4)		50(32.5)	51(30.5)	62(31.6)	
Strong	133(31.3)	13(16.9)		15(9.7)	44(26.3)	89(45.4)	
missing	13	1		5	3	2	
**P21 fraction**							
0-1%	69(16.3)	3(3.9)	0.002**	25(16.2)	27(16.2)	25(12.9)	0.835
2-25%	233(55.1)	41(53.2)		81(52.6)	90(53.9)	112857.7)	
26-50%	60(14.2)	18(23.4)		24(15.6)	28(16.8)	26(13.4)	
51-75%	53(12.5)	13(16.9)		21(13.6)	16(9.6)	30(15.5)	
>75%	8(1.9)	2(2.6)		3(1.9)	6(3.6)	1(0.5=	
missing	15	1		5	3	4	
**P21 intensity**							
Negative	69(16.3)	3(3.9)	<0.001**	25(16.2)	27(16.2)	25(12.9)	0.567
Weak	174(41.1)	24(31.2)		60(39.0)	63(37.7)	81(41.8)	
Moderate	122(28.8)	36(46.8)		51(33.1)	52(31.1)	60(30.9)	
Strong	58(13.7)	14(18.2)		18(11.7)	25(15.0)	28(14.4)	
missing	15	1		5	3	4	
**MSI screening status**							
MSS	-	-		109(72.7)	136(83.4)	184(95.8)	<0.001**
MSI	-	-		41(27.3)	27(16.6)	8(4.2)	
missing				9	7	6	

Columnar percentage in parentheses. χ2-test of association was used for 2x2 tables and χ2-test for linear trend for tables with more than two rows and/or columns. The categories marked as missing were not included in the analysis. *Significant at the 0.05 level. **Significant at the 0.01 level. MSS = Microsatellite stable, MSI = Microsatellite unstable.

### Association of MSI screening status and beta-catenin overexpression with survival

**Figure 2:**
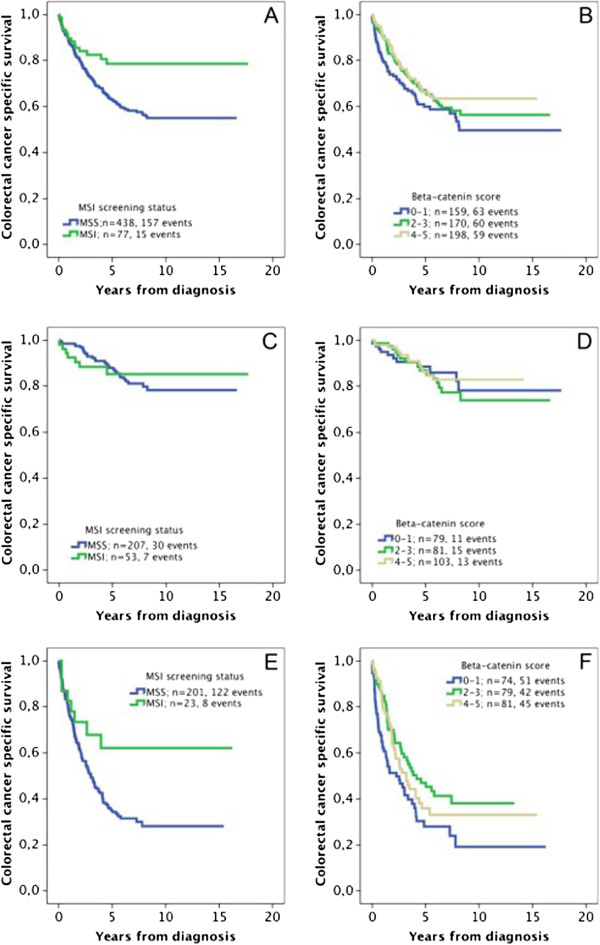
**Associations between MSI screening status, beta-catenin overexpression and survival in all patients and in subgroups according to disease stage.** Kaplan-Meier analysis of colorectal cancer specific survival according to: (**A**) MSI screening status in the full cohort (logrank p = 0.008**), (**B**) Beta-catenin score in the full cohort (logrank p = 0.116), (**C**) MSI screening status in stage I-II disease (logrank p =0.985), (**D**) Beta-catenin score in stage I-II disease (logrank p =0.725), (**E**) MSI screening status in stage III-IV disease (logrank p =0.032*) and (**F**) Beta-catenin score in stage III-IV disease (logrank p =0.034*).

Kaplan-Meier analysis showed significant correlations between MSI and prolonged CSS in the whole cohort (Figure 2A), and in stage III-IV disease (Figure 2E) but not in stage I-II disease (Figure 2C). Beta-catenin overexpression correlated significantly to a prolonged CSS in stage III-IV disease only (Figure 2F).

Uni- and multivariable (adjusted for age, T-stage, N-stage, M-stage, differentiation grade and vascular invasion) Cox regression analysis confirmed the significant correlation between MSI and an improved CSS, both in the full cohort and in stage III-IV disease (Table 3). Beta-catenin overexpression was also associated with an improved CSS, in the full cohort and patients with stage III-IV disease, although these associations were less significant than for MSI status (Table 3). Notably, while only reaching statistical significance in multivariable analysis, the HRs for beta-catenin overexpression should be considered as being similar in univariable and multivariable analysis (0.75 vs 0.70 in the full cohort and 0.73 vs 0.67 in stage III-IV disease).

**Table 3:** Cox proportional hazards model for colorectal cancer specific survival related to MSI screening status and beta-catenin overexpression in the full cohort and in stage III-IV disease

	**Full cohort**			**Stage III-IV disease**		
	**HR(95% CI)**	***p-value***	***N(events)***	**HR(95% CI)**	***p-value***	***n(events)***
		*Univariable*			*Univariable*	
MSS	1,00	0,010**	438(157)	1,00	0,036*	201(122)
MSI	0,50(0,29-0,84)		77(15)	0,46(0,23-0,95)		23(8)
		*Multivariable*			*Multivariable*	
MSS	1,00	0,010*	389(137)	1,00	0,011*	185(107)
MSI	0,46(0,25-0,84)		73(13)	0,33(0,14-0,78)		20(6)
		*Univariable*			*Univariable*	
Bata-catenin low	1,00	0,056	251(96)	1,00	0,063	112(72)
Bata-catenin High	0,75(0,56-1,01)		276(86)	0,73(0,52-1,02)		122(66)
		*Multivariable*			*Multivariable*	
Bata-catenin low	1,00	0,031*	223(80)	1,00	0,036*	98(60)
Bata-catenin High	0,70(0,51-0,97)		247(75)	0,67(0,46-0,97)		113(57)

*Significant at the 0.05 level. **Significant at the 0.01 level. P-value from multivariable analysis adjusted for age (>/<=75 years), gender, T-stage (I-II, III, IV), N-stage (0,1,2), M-stage (0, 1), differentiation grade (high-intermediate vs low) and vascular invasion (+/−/unknown). Beta-catenin low = beta-catenin score 0–2. Beta catenin high = beta-catenin score 3–5.

**Figure 3:**
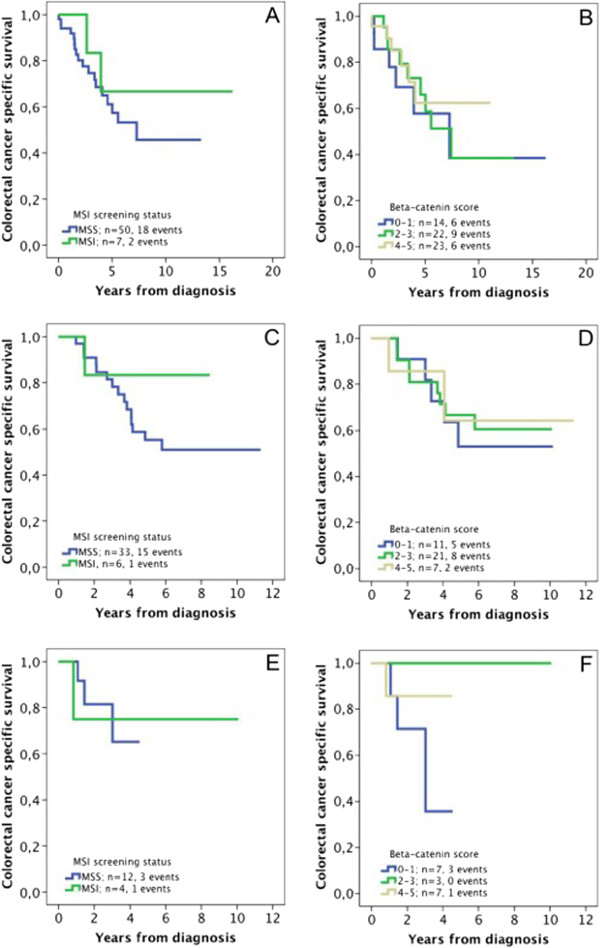
**Associations between MSI screening status, beta-catenin expression and survival in stage III disease stratified according to adjuvant therapy.** Kaplan-Meier analysis of colorectal cancer specific survival according to (**A**) MSI screening status and no adjuvant treatment (logrank p = 0.415), (**B**) Beta-catenin score and no adjuvant treatment (logrank p = 0.783), (**C**) MSI screening status and adjuvant FLV (5-fluorouracil + leucovorin ) (logrank p = 0.251), (**D**) Beta-catenin score and adjuvant FLV (logrank p = 0.927), (**E**) MSI screening status and adjuvant FLOX (5-fluorouracil + leucovorin + oxaliplatin ) (logrank p = 0.871) and (**F**) Beta-catenin score and adjuvant FLOX (logrank p = 0.336).

Next, we examined the potential treatment predictive value of MSI screening status and beta-catenin overexpression. Subgroup analysis of stage III disease patients in strata according to adjuvant treatment (no treatment, 5-fluorouracil + leucovorin or 5-fluorouracil + leucovorin + oxaliplatin) revealed no significant correlations of MSI or beta-catenin overexpression and CSS (Figure 3).

The prognostic and treatment predictive value of MSI and beta-catenin overexpression did not differ according to gender (data not shown). Similar trends for the prognostic and treatment impact of MSI status and beta-catenin expression were seen for 5-year overall survival (data not shown).

## Discussion

In this study we have demonstrated that positive MSI screening status is associated with a more favourable prognosis and certain clinicopathological characteristics of CRC, i.e. older age, female sex, proximal tumour location and low differentiation grade. This is accordant to previous studies [16,17], thus consolidating our data. The correlation between MSI and a prolonged CSS was significant in both uni- and multivariable analysis for the whole cohort and for stage III-IV disease, but not for stage I-II disease. A possible interpretation of this observation could be that MSI reflects a less agressive phenotype, with an improved CSS even in the presence of metastatic disease and despite adverse pathological characteristics such as low differentiation grade.

Here, we could not demonstrate any association between MSI status and response to adjuvant treatment in curatively treated patients with stage III disease. On one hand, the subgroups available for analysis of the effects of adjuvant treatment were rather small, thus limiting statistical power. On the other hand, since this study started already in the mid 90s, when adjuvant chemotherapy was not yet standard of care in Sweden, nearly half of the curatively treated patients with stage III disease did not receive adjuvant treatment. Although treatment predictive markers are best evaluated in a controlled, prospective setting, the value of retrospective analyses on tumours from less extensively treated patients should not be underestimated.

Moreover, the feasibility of MSI as a predictor of therapeutic response has been debated, and a recent meta-analysis showed results concordant with ours, i.e. no treatment predictive value of MSI status [34].

Beta-catenin overexpression was also associated with a reduced risk of death in the full cohort and in patients with stage III-IV disease, although these associations were weaker than for MSI status. Previous studies on beta-catenin as a prognostic marker have been conflicting, with some studies reporting a favourable or no prognostic value [14,35-38] and some an association with poor clinical outcome [12,13,39,40]. Many factors could explain the differing results regarding the prognostic value of beta-catenin overexpression; e.g. intrinsic tumour heterogeneity [41], different immunohistochemical staining and visualization methods with varying degrees of sensitivity, and lack of standardization of what constitutes a “positive” or “negative” result. In many cases, nuclear beta-catenin expression is predominantly seen in the margin and not in the center of the tumour [42]. Horst et al. postulated that lost beta-catenin regulation is presented rather as an altered intratumoral distribution of nuclear beta-catenin expression [43] and, in light of this observation, the TMA-technique may not be an ideal tool for its assessment. Moreover, the degree and prognostic implications of beta-catenin activation may differ in primary and metastatic CRC [44].

The herein observed inverse association between MSI and beta-catenin overexpression is also in accordance with previous studies [45,46]. Assuming that MSI status and beta-catenin overexpression represent two different pathways to colorectal tumorigenesis, it might be perceived as contradictory that both reflect an improved CSS. However, as these pathways are not mutually exclusive and likely overlap, their complex interactions need further study.

The associations between MSI and cell cycle regulators cyclin D1 and p21 have been described previously [47,48] but the underlying mechanisms still remain unclear. Similarly, the inverse associations of MSI tumours with p53 mutation status and p27 expression are well known [30,48]. The positive correlation between beta-catenin overexpression and p53 status is also in line with previous studies [14,39,45].

The observation of a positive association of cyclin D1 expression with MSI screening status and beta-catenin overexpression, respectively, is in line with the previously demonstrated findings of cyclin D1 expression being associated with a prolonged CSS [26]. Notably, the beneficial prognostic value of high cyclin D1 expression was only evident in male and not in female CRC patients [26]. In this study, the prognostic and treatment predictive value of MSI and beta-catenin overexpression did not differ according to sex.

Since the MDCS is a population-based cohort study, a potential selection bias compared with the general population must be taken into consideration, but the distribution of TNM-stages at diagnosis is in line with the expected, with no predilection for less advanced stages [26,28]. Notably, while the distribution of clinicopathological factors and survival from the disease did not differ between women and men [26], a recent study demonstrated sex-related differences in the associations of anthropometric factors with CRC risk by TNM stage and tumour location in the present cohort [49]. These findings provide further evidence for colorectal cancer being a disease in which the influence of sex hormones should not be underestimated. Therefore, the prognostic and treatment predictive value of investigative CRC biomarkers should always be considered in strata according to gender. Moreover, it will be of interest to investigate the influence of anthropometric, hormonal and life-style factors on risk of CRC defined by MSI status and beta-catenin alterations in the present cohort.

Since the number of events was identical for CRC-specific and overall survival in patients with metastatic disease, as previously demonstrated [26], the use of CRC-specific survival as primary endpoint in the survival analyses in this study should be a reasonable surrogate for cancer-specific outcome. Moreover, similar survival trends were observed for 5-year overall survival.

## Conclusions

In conclusion, the findings from this large cohort study demonstrate that MSI screening status in colorectal cancer is an independent prognostic factor, but not in localized disease, and does not predict response to adjuvant chemotherapy. Despite its inverse correlation with MSI status, we also observed a link between beta-catenin and prolonged survival from colorectal cancer. Associations of MSI and beta-catenin alterations with other investigative and clinicopathological factors were largely in line with the expected.

## Abbreviations

CRC: Colorectal cancer; MSI: Microsatellite instability; MDCS: Malmö diet and cancer study.

## Competing interests

The authors declare that no competing interests exist.

## Authors’ contributions

SW collected clinical data, evaluated the immunohistochemical stainings, performed the statistical analyses and drafted the manuscript. JB assisted with the collection of clinical data and helped draft the manuscript. BN constructed the TMAs and carried out all IHC stainings. KEL participated in the evaluation of the immunohistochemical staining. KJ conceived of the study, carried out the histopathological re-evaluation, evaluated the immunohistochemistry, and helped draft the manuscript. JE conceived of the study, assisted with the collection of clinical data, and helped draft the manuscript. All authors read and approved the final manuscript.
